# γ-Tocotrienol inhibits HeLa cell proliferation likely via modulation of the PI3K/AKT/mTOR signaling pathway

**DOI:** 10.3389/fnut.2026.1804277

**Published:** 2026-04-16

**Authors:** Yichun Tian, Xian Wang, Zhaoxin Lu, Yaqing Mi, Shaobo Zhou, Weili Xu

**Affiliations:** 1 Innovation Research Center for Special Food-Medicine and Biochemical Engineering, School of Chemistry and Chemical Engineering, Harbin Institute of Technology, Harbin, China; 2 School of Science, Faculty of Engineering and Science, University of Greenwich, Central Avenue, Chatham, United Kingdom

**Keywords:** γ-tocotrienol, cervical cancer, proliferation, apoptosis, PI3K/AKT/mTOR signaling pathway

## Abstract

**Introduction:**

γ-Tocotrienol (γ-T3), a natural isoform of vitamin E, has demonstrated anticancer activity; however, its underlying molecular mechanisms remain incompletely understood. This study investigated whether γ-T3 suppresses human cervical cancer HeLa cell growth through modulation of the phosphatidylinositol 3-kinase/protein kinase B/mammalian target of rapamycin (PI3K/AKT/mTOR) signaling pathway.

**Methods:**

HeLa cells were treated with γ-T3 at different concentrations (0-80 μmol/L). Protein expression and phosphorylation levels of PI3K, AKT, mTOR and downstream effectors (p70S6K and 4E-BP1) were analyzed. Cell proliferation, cell cycle distribution and apoptosis were assessed. Wortmannin (WM), a selective PI3K inhibitor, was used as a comparator. Combined treatment with γ-T3 and WM was also evaluated.

**Results:**

γ-T3 treatment reduced the expression and phosphorylation of PI3K, AKT and mTOR, as well as downstream targets p70S6K and 4E-BP1. γ-T3 also decreased proliferation-associated proteins cyclin D1 and c-Myc. The inhibitory effect of γ-T3 at 40 μmol/L was comparable to that of WM. Functionally, γ-T3 suppressed cell proliferation, induced G0/G1 phase arrest with a reduced S-phase fraction, and promoted apoptosis in HeLa cells. Co-treatment with γ-T3 and WM further enhanced growth inhibition and apoptosis compared with either treatment alone.

**Discussion:**

These findings indicate that γ-T3 inhibits HeLa cell proliferation, at least in part, via suppression of the PI3K/AKT/mTOR signaling pathway. This supports further evaluation of γ-T3 as a nutrition-relevant bioactive compound for cancer prevention research and as a potential adjunct to therapy.

## Highlights

- γ-Tocotrienol inhibits PI3K/AKT/mTOR signaling and downstream effectors in HeLa cells.
- At 45 μmol/L, γ-tocotrienol shows pathway inhibition comparable to the PI3K inhibitor wortmannin.
- γ-Tocotrienol suppresses HeLa cell proliferation by inducing G0/G1 arrest and apoptosis.
- Co-treatment with γ-tocotrienol and wortmannin enhances anti-proliferative effects compared with either agent alone.

## Introduction

Cervical cancer is one of the most common gynecological malignancies and remains an important cause of cancer-related mortality among women worldwide (1). Although advances in surgery, chemotherapy and radiotherapy have improved clinical outcomes, treatment-related toxicity and limited efficacy in advanced disease highlight the need for safer and more effective therapeutic strategies (2, 3). Bioactive compounds derived from dietary sources have attracted increasing attention because of their potential roles in cancer prevention and as adjunct therapeutic agents (4–6).

Vitamin E is a group of fat-soluble compounds composed of two major categories: tocopherols and tocotrienols (T3), each including four isoforms (α, β, γ, δ), resulting in eight structurally related molecules. All vitamin E isoforms share a chromanol ring structure attached to a hydrophobic side chain at the 2-position. Compared with tocopherols, T3 possess an unsaturated isoprenoid side chain containing three double bonds, which may enhance membrane penetration and influence biological activity (7). Although T3 have been reported to exhibit strong antioxidant capacity (8), increasing evidence suggests that their anticancer activity is not solely attributable to antioxidant properties but may also be related to the structural characteristics of the chromanol ring and unsaturated side chain (9–11).

Compared with tocopherols, T3 has been suggested to exhibit distinct biological activities and may provide additional therapeutic value in cancer prevention strategies (12–14). Extensive reviews have summarized the anticancer properties of T3, including regulation of cell proliferation, apoptosis, oxidative stress, inflammation, and signaling pathways involved in tumor development (7, 12–17). Among the T3 isoforms, γ-T3 has attracted particular attention because of antitumour activity in several cancer models, including hepatocellular carcinoma (18, 19), gastric cancer (20), colorectal cancer (21), and breast cancer (22). γ-T3 has been reported to selectively target cancer cells, exhibiting no toxicity or extremely low toxicity to normal cells at treatment doses (23–25). Importantly, human observational evidence has demonstrated the presence of T3 in adipose tissues of patients with benign and malignant breast tumors, supporting their bioavailability and physiological relevance in humans (26). Although clinical evidence remains limited, current data suggest that T3 are biologically active *in vivo* and may contribute to cancer preventive effects through modulation of multiple molecular targets. Furthermore, γ-T3 occurs naturally in dietary sources such as palm oil and rice bran oil (27). These findings support further investigation of γ-T3 as a nutraceutical compound with potential cancer chemopreventive relevance. Nevertheless, the molecular mechanisms underlying its effects remain incompletely understood.

Emerging evidence indicates that the phosphatidylinositol-3-kinase/protein kinase B/mammalian target of rapamycin (PI3K/AKT/mTOR) signaling pathway is a key regulator of cell growth, metabolism, and migration in multiple cancers (28–31). Abnormal activation of this pathway is frequently observed in various malignancies, including cervical cancer (31–36). Consequently, targeting PI3K/AKT/mTOR has become an important therapeutic strategy, and PI3K inhibitors are advancing through preclinical and clinical development (37–39). Notably, γ-T3 has been reported to suppress PI3K/AKT activity, leading to antiproliferative and autophagy-inducing effects in highly malignant +SA mammary epithelial cells (40–43).

In cervical cancer CaSki cells, the antiproliferative effect of γ-T3 has been linked to reduced expression of MEK-2 and ERK-2 (44). Our previous findings further showed that γ-T3 suppresses cervical cancer cell growth and induces apoptosis via the mitochondrial pathway (45). However, whether γ-T3 regulates PI3K/AKT/mTOR signaling in cervical cancer HeLa cells has not been fully elucidated. Here, we investigated the effects of γ-T3 on PI3K/AKT/mTOR pathway activity and evaluated associated functional outcomes, including cell viability, cell-cycle distribution and apoptosis. We further compared γ-T3 with wortmannin (WM) and assessed the effects of combined treatment. This work provides mechanistic evidence supporting γ-T3 as a nutrition-relevant bioactive compound worthy of further investigation.

## Materials and methods

### Reagents

γ-T3 (Cayman, Ann Arbor, MI, USA) was dissolved in absolute ethanol to prepare a 2 × 10^4^ μmol/L stock solution and stored at −20 °C. The following experimental reagents were used: modified RPMI-1640 medium (Thermo Fisher Scientific, Beijing, China), fetal bovine serum (Tianhang Biotech, Zhejiang, China), trypsin (Amresco, USA), PBS buffer (Solarbio, Beijing, China), WM (Solarbio, Beijing, China), MTT (Sigma Aldrich, Kansas, MO, USA), DMSO (Fuyu Chemical, Tianjin, China), SDS-PAGE gel kit (Applygen, Beijing, China), BCA protein assay kit (Applygen, Beijing, China), PMSF (Applygen, Beijing, China), RIPA cell lysis buffer (Applygen, Beijing, China), Cell cycle and apoptosis detection kit (Beyotime Biotech, Shanghai, China), Hoechst 33342/PI staining kit (Solarbio, Beijing, China), alkaline phosphatase chromogenic solution (Promega Corporation, Madison, WI, USA), and nitrocellulose membrane (Pall Corporation, New York, USA). The following antibodies were used: anti-mTOR, anti-cyclin D1, anti-c-Myc (Cell Signaling), anti-GAPDH, anti-PCNA, anti-PI3K, anti-AKT, anti-p-AKT (Ser473), anti-p-mTOR (Ser2448), anti-p70S6K, anti-p-p70S6K, anti-4E-BP1, anti-p-4E-BP1 (Santa Cruz Biotechnology, Dallas, TX, USA), as well as horseradish peroxidase (HRP)-labeled goat anti-rabbit IgG and goat anti-mouse IgG (Santa Cruz Biotechnology, Dallas, TX, USA).

### Human cell lines

Human cervical cancer HeLa cells were provided by the Department of Pathophysiology, Basic Medical College of Jiamusi University (Heilongjiang, China) and cultured in RPMI-1640 medium supplemented with 10% fetal bovine serum in an atmosphere containing 5% CO_2_ at 37 °C.

### Cell viability assay and morphology observations

Cell viability was assayed using the MTT method. HeLa cells in the logarithmic growth phase were digested with 0.25% trypsin and prepared as cell suspensions. The cells were seeded at a density of 2 × 10^4^ cell/well in 24-well plates and cultured overnight. The medium was then replaced with fresh medium containing 5% fetal bovine serum mixed with different concentrations of γ-T3 (0, 15, 30, 45, and 60 μmol/L). Blank control and solvent control groups were included, with three parallel wells each. After incubation for the designated time, 100 μL of MTT solution (5 mg/mL) was added to each well and incubated for 4 h. The supernatant was then discarded, and 750 μL of DMSO was added to each well. The plates were agitated at 140 rpm on an oscillating shaker for 10 min to dissolve the formazan crystals. Absorbance (OD) was measured at 490 nm using a Model 550 microplate reader (Bio-Rad, Hercules, CA, USA). Morphological changes of HeLa cells in both control and treated groups were observed under an inverted optical microscope (EVOS XL Core, Thermo Scientific, USA).

### Hoechst 33342/PI double-staining

After fixation, the supernatant was discarded, and the cell slides were washed with PBS. Then, 100 μL of cell staining buffer, 5 μL of Hoechst 33342 solution, and 5 μL of PI solution were added sequentially. The samples were incubated in the dark at 4 °C for 20–30 min, washed with PBS, and observed under a fluorescence microscope (Olympus IX70, Tokyo, Japan).

### Cell cycle and apoptosis analysis

HeLa cells in the logarithmic growth phase were seeded at a density of 8.5 × 10^5^ cells/dish in 60-mm culture dishes and incubated overnight. The culture medium was then replaced with fresh medium containing 5% fetal bovine serum mixed with 45 μmol/L γ-T3 and/or 3 μmol/L WM, and the cells were incubated for 24 h. Both suspension and adherent cells were collected into 1.5 mL Eppendorf tubes, fixed with 1 mL of 70% ethanol (precooled to −20 °C), and stored for 24 h at 4 °C. The cells were pelleted by centrifugation at 1,000 rpm for 5 min, the supernatant discarded, and the cells resuspended in 1 mL of ice-cold PBS. Samples were analyzed by flow cytometry (Guava easyCyte Mini System, Millipore, USA) using the Cell Cycle and Apoptosis Detection Kit (Beyotime Biotech, Shanghai, China). Data were processed using ModFit LT software (Verity Software House, Topsham, ME, USA).

### Western blot analysis

Suspension and adherent cells were collected into 1.5 mL Eppendorf tubes and lysed with 50 μL of lysis buffer on ice for 20 min. The lysates were centrifuged at 12,000 rpm for 10 min at 4 °C, and the supernatants were collected. Protein concentrations were determined and normalized using a BCA protein assay kit (Applygen, Beijing, China). Equal amounts of protein were loaded, separated by SDS-PAGE, and transferred to NC membranes (45). Protein bands were visualized using chromogenic reagent kits, photographed with the FluorChem Imaging System (Bio-Rad, USA), and analyzed by densitometry with Quantity One software.

### Statistical analysis

All data were processed and plotted using Microsoft Excel 2016. Results are expressed as mean ± SD from at least three independent experiments unless stated otherwise. Group differences were analyzed by one-way analysis of variance (ANOVA) followed by Duncan's multiple range test for *post-hoc* comparisons. *P* < 0.05 was considered statistically significant.

## Results

### Effect of γ-T3 on the viability and apoptosis of HeLa cells

**Figure 1:**
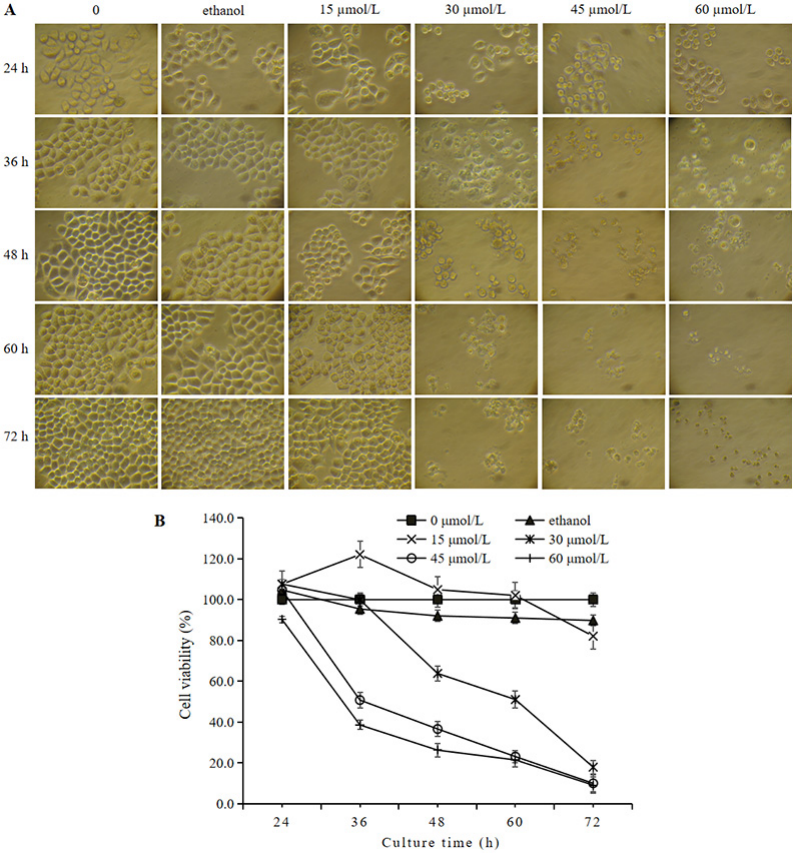
Effect of γ-T3 on the morphology and viability of HeLa cells. **(A)** Morphological observation of HeLa cells in control group and experimental groups under an inverted microscope (200×). **(B)** Effect of γ-T3 on the viability of HeLa cells. HeLa cells were treated with the different concentrations of γ-T3 for 24, 36, 48, 60, and 72 h and examined by the MTT method. Triplicates were used (*n* = 3). Data were presented as the mean ± SD.

The morphological changes of HeLa cells treated with γ-T3 for the designated times are presented in Figure 1A. The cells in the blank control and solvent groups showed relatively large rhombic or irregular shapes. Other changes included narrowing of intercellular spaces and an increase in the number of HeLa cells with incubation time. A low concentration of γ-T3 (15 μmol/L) had no significant effect on the morphology of HeLa cells. By contrast, HeLa cells treated with higher concentrations (30–60 μmol/L) of γ-T3 exhibited shrinkage, size reduction, rounding and separation from each other. These phenomena became more pronounced with increasing γ-T3 doses and longer incubation times.

The effect of γ-T3 on HeLa cell viability was further investigated using the MTT assay. As shown in Figure 1B, cell viability in the solvent control group was not affected compared with the blank control group. γ-T3 (30–60 μmol/L) inhibited HeLa cell viability in a dose- and time-dependent manner. The inhibition rates of HeLa cells treated with 30, 45, and 60 μmol/L γ-T3 for 48 h were 36.2%, 63.3%, and 73.7%, respectively. The 50% inhibitory concentration (IC50) of γ-T3 was 37.4 ± 5.7 μmol/L at 48 h.

**Figure 2:**
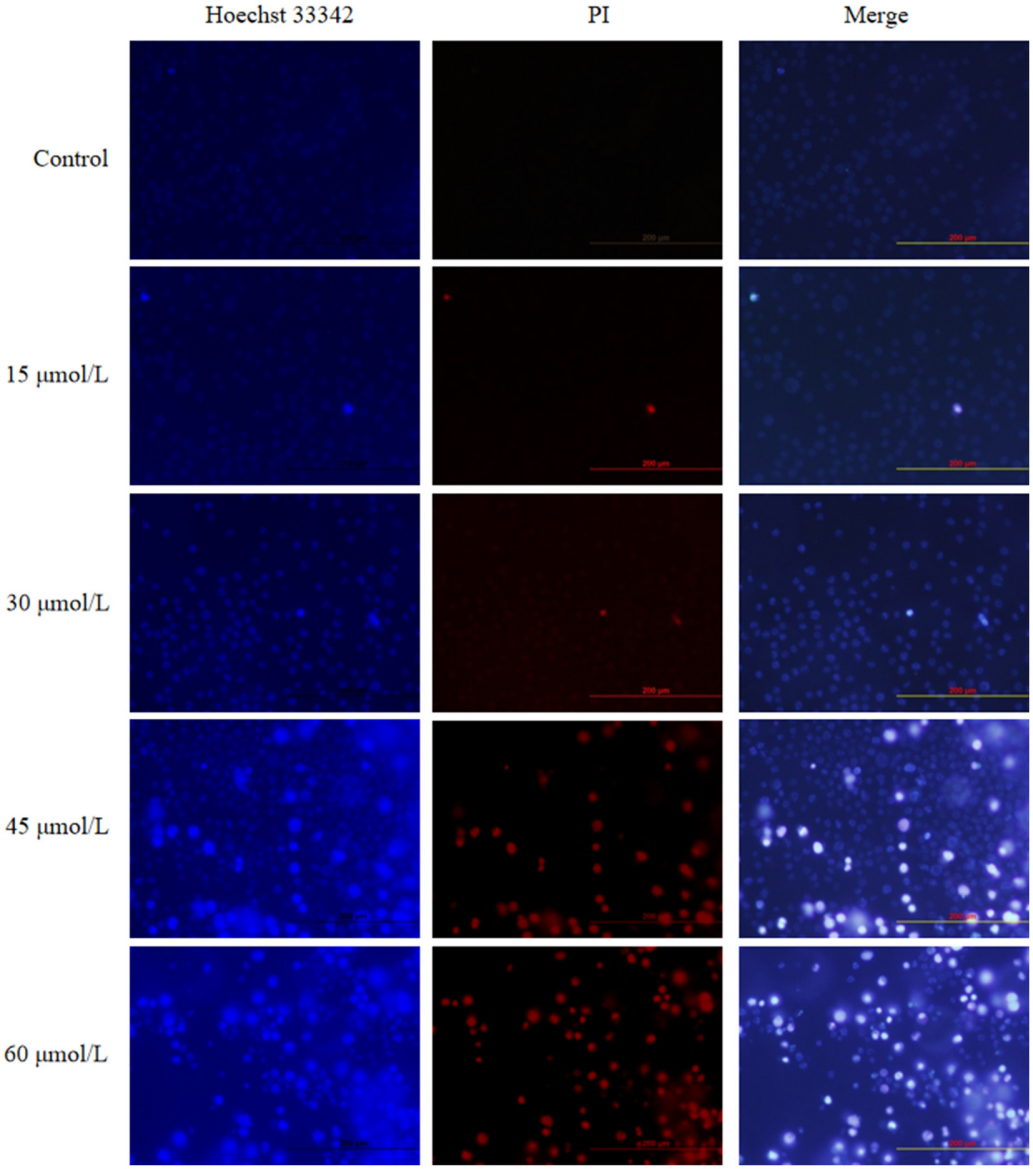
Effect of γ-T3 on the apoptosis of HeLa cells. Cells were treated with 15, 30, 45, and 60 μmol/L of γ-T3 for 24 h and examined using Hoechst 33342 and PI staining (×200).

Hoechst 33342 and PI staining were performed to investigate the apoptotic effect of HeLa cells treated with different concentrations of γ-T3 for 24 h. The nuclei of apoptotic cells showed chromatin condensation. Hoechst 33342 dye penetrated the cell membrane, and apoptotic cells displayed stronger blue fluorescence than normal cells. PI dye, which does not penetrate intact cell membranes, stained the nuclei of apoptotic and necrotic cells due to membrane damage (46). As shown in Figure 2, cells in the blank control and solvent groups (not shown) exhibited weak blue and very weak red fluorescence. No significant changes were observed in cells treated with 15 μmol/L γ-T3. However, cells treated with 30 and 60 μmol/L γ-T3 displayed a dose-dependent increase in apoptotic cells (weak red and strong blue fluorescence) and necrotic cells (strong red and strong blue fluorescence). These results indicate that γ-T3 could induce apoptosis in HeLa cells.

### γ-T3 downregulates the PI3K/AKT/mTOR signaling pathway in human cervical HeLa cells

**Figure 3:**
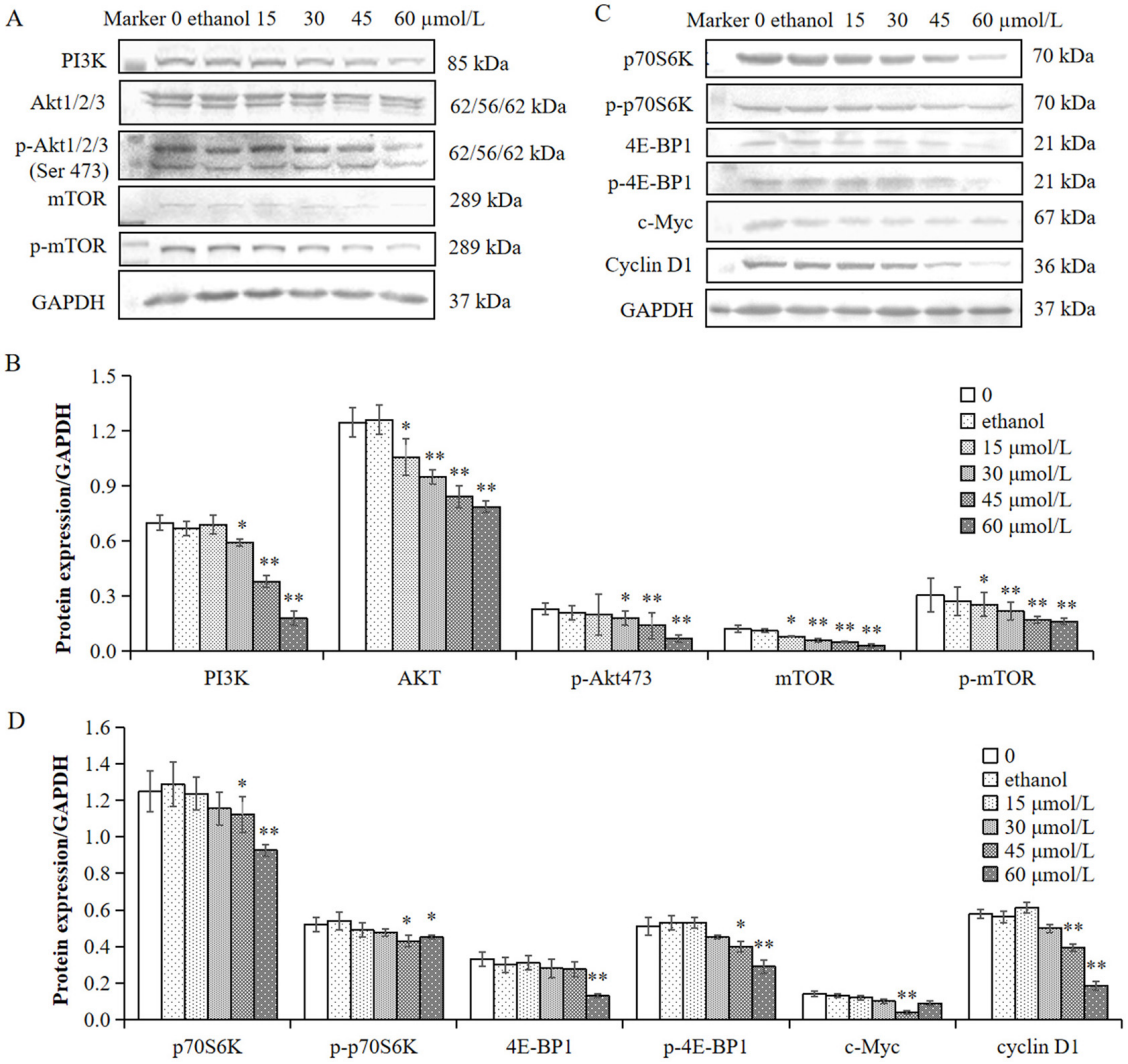
Effect of γ-T3 on the PI3K/AKT/mTOR pathway in HeLa cells. Expression levels of PI3K, (+/−p) AKT, (+/−p) mTOR, which are shown in **(A, B)**, and (+/−p) p70S6K, (+/−p) 4E-BP1, c-Myc, and cyclin D1, which are shown in **(C, D)**, in HeLa cells treated with γ-T3 at 0, 15, 30, 45, and 60 μmol/L and ethanol for 24 h were detected by Western blot. Values are presented as the mean ± SD (*n* = 3). ^*^P < 0.05, ^**^P < 0.01 vs. 0 μmol/L γ-T3 (one-way ANOVA with Duncan's multiple range test).

Many natural plant-derived chemicals and active constituents used in food or medicine can exert antitumour effects by inhibiting the PI3K/AKT/mTOR pathway (47–53). To investigate whether γ-T3 regulates this pathway in HeLa cells, the expression of upstream proteins (PI3K, (+/–p) AKT, and (+/–p) mTOR), downstream proteins ((+/–p) p70S6K and (+/–p) 4E-BP1), and target proteins (c-Myc and cyclin D1) was examined using Western blot. As shown in Figure 3, treatment with 30–60 μmol/L γ-T3 significantly downregulated PI3K, (+/–p) AKT, (+/–p) mTOR, (+/–p) p70S6K, (+/–p) 4E-BP1, c-Myc, and cyclin D1 compared with the blank control group, particularly at 45 and 60 μmol/L (*P* < 0.05 or *P* < 0.01). No significant differences were observed among the 15 μmol/L γ-T3 group, solvent control group, and blank control group. These results indicated that 30–60 μmol/L γ-T3 could inhibit the PI3K/AKT/mTOR signaling pathway in human cervical cancer HeLa cells.

### Effect of WM on the γ-T3-inhibited PI3K/AKT/mTOR signaling pathway

**Figure 4:**
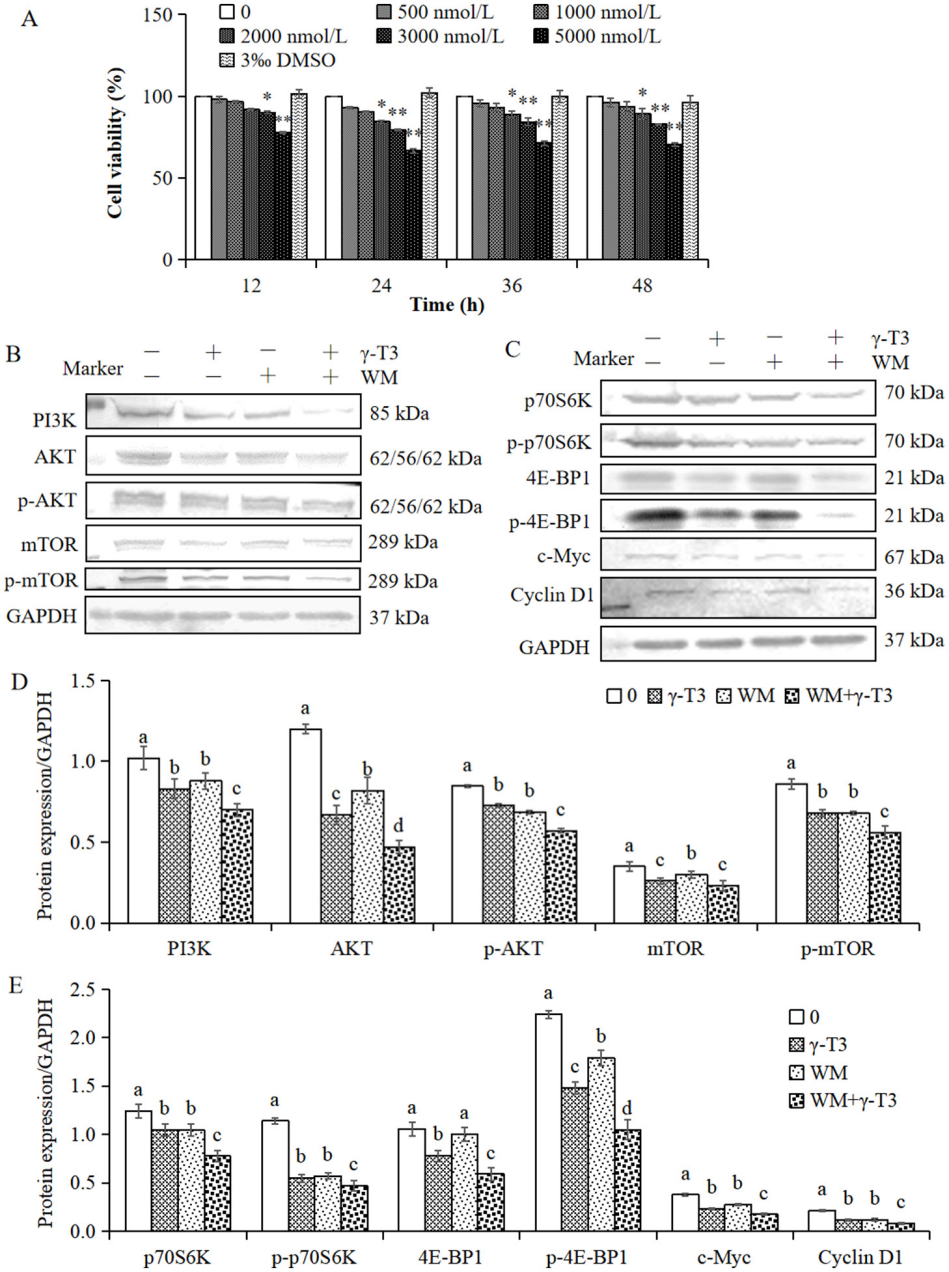
Effect of WM on γ-T3-inhibited PI3K/AKT/mTOR signaling in HeLa cells. **(A)** Effect of different concentrations of WM on cell viability. HeLa cells were treated with 500, 1,000, 2,000, 3,000, and 5,000 nmol/L WM for 12, 24, 36, and 48 h. Values are presented as the mean ± SD (*n* = 3). *P* < 0.05; ^*^P < 0.01 vs. 0 nmol/L WM (one-way ANOVA with Duncan's multiple range test). Expression of PI3K, (+/−p) AKT, (+/−p) mTOR **(B)**, (+/−p) p70S6K, (+/−p) 4E-BP1, c-Myc, and cyclin D1 **(C)** determined by Western blot after treatment with γ-T3 (45 μmol/L), WM (3 μmol/L) or their combination for 24 h. Values are presented as the mean ± SD (*n* = 3). Different letters indicate significant differences (*P* < 0.05, one-way ANOVA with Duncan's multiple range test) **(D, E)**.

The PI3K selective inhibitor WM was introduced to examine the specificity of γ-T3 on the PI3K/AKT/mTOR pathway (54). The morphology and viability of HeLa cells treated with different doses of WM for 12, 24, 36, and 48 h were measured to determine the appropriate dose. As shown in Figure 4A, WM at 500–5,000 nmol/L inhibited HeLa cell growth in a dose-dependent manner. Within 12–24 h, the inhibition rate increased with WM concentration; however, no further significant increase was observed at 36–48 h. The growth inhibition rates of HeLa cells treated with 2,000, 3,000, and 5,000 nmol/L WM for 24 h were 15.67%, 20.68%, and 33.45%, respectively, which were significantly different from the blank control group (*P* < 0.01). Therefore, 3 μmol/L WM was selected for subsequent experiments.

HeLa cells treated with 45 μmol/L γ-T3 and 3 μmol/L WM alone or in combination for 24 h were examined by Western blot. As shown in Figures 4B–E, WM significantly decreased the expression of PI3K, ±p-AKT, ±p-mTOR, ±p-p70S6K, ±p-4E-BP1, c-Myc, and cyclin D1 compared with the control group, with effects similar to those of γ-T3. Furthermore, WM enhanced γ-T3-induced downregulation of the PI3K/AKT/mTOR pathway. These results support the conclusion that 45 μmol/L γ-T3 inhibits the PI3K/AKT/mTOR pathway, which contributes to its inhibitory effects on HeLa cells.

### Effect of the combination of WM and γ-T3 on the viability of HeLa cells

**Figure 5:**
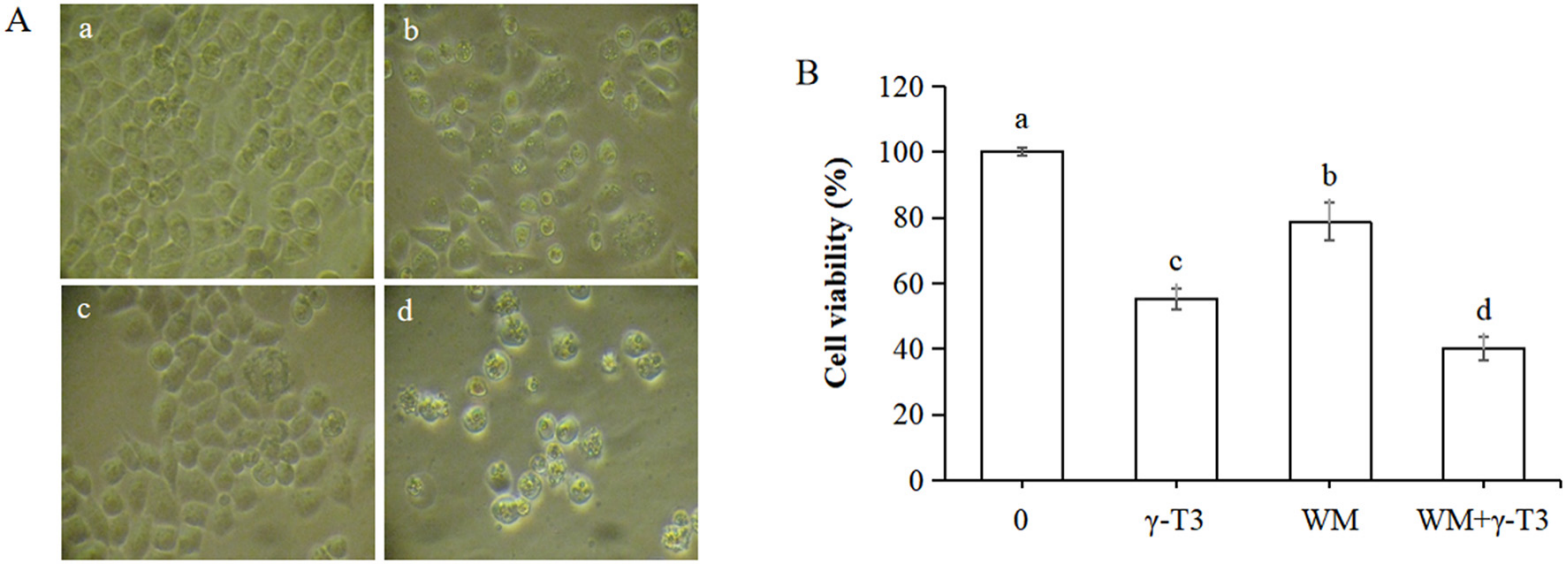
Effect of WM and γ-T3 alone or in combination on the morphology and viability of HeLa cells. **(A)** Morphological changes of HeLa cells treated by WM and γ-T3 (under an inverted microscope, 200×) for 24 h. **(a)**, blank control group; **(b)**, γ-T3 (45 μmol/L); **(c)**, WM (3 μmol/L); and **(d)**, WM (3 μmol/L)+ γ-T3 (45 μmol/L). **(B)** Effect of WM and γ-T3 on HeLa cell viability. HeLa cells were treated by γ-T3 at 45 μmol/L and WM at 3 μmol/L for 24 h and examined by the MTT method. Values are presented as the mean ± SD (*n* =3). Different letters indicate significant differences (*P* < 0.05).

The morphological differences of HeLa cells treated with 45 μmol/L γ-T3 and 3 μmol/L WM alone or in combination for 24 h were examined under an inverted microscope (Figure 5A). Cells in the control group were adherent, large, irregularly shaped, and closely packed with abundant cytoplasm (Figures 5A, a). In the γ-T3 group, cell numbers decreased, intercellular spaces widened, and some cells detached and floated (Figures 5A, a, b). In the WM group, a few cells became round and shrunken, though fewer than in the control (Figures 5A, a, c). In the combined treatment group, cells were smaller and round, with significantly reduced numbers. Adherence was weakened, nuclei shrank, and some cells ruptured. Necrotic/dead cells increased and floated in the medium (Figures 5A, a, d).

Cell viability was also assessed using the MTT assay. As shown in Figure 5B, the viability rates of HeLa cells in the γ-T3, WM, and combined groups were 55.23% ± 3.33%, 78.73% ± 5.84%, and 40.17% ± 3.60%, respectively, indicating that the combined treatment resulted in the lowest cell viability among the tested groups. Statistically significant differences (*P* < 0.05) were found among the control, γ-T3, WM, and combined groups. These results indicate that co-treatment with WM further reduced cell viability compared with γ-T3 alone, suggesting an enhanced inhibitory effect when both agents were applied together, consistent with the morphological observations.

### Effect of the combination of WM and γ-T3 on the cell cycle of HeLa cells

Cell cycle progression consists of interphase and the mitotic (M) phase. Interphase is divided into G1 (DNA synthesis preparation), S (DNA synthesis), and G2 (post-DNA synthesis) phases (55, 56). Malignant tumor development is often associated with cell cycle dysregulation (57), and tumor cells typically show a higher proportion of S -phase cells and proliferative activity than normal cells (58).

Flow cytometry was performed to examine the effect of γ-T3 on cell cycle distribution in HeLa cells and to explore the role of the PI3K/AKT/mTOR pathway (Table 1). The percentage of G0/G1 phase cells increased from 54.52% ± 1.97% (control) to 72.36% ± 4.65% (γ-T3), 64.21% ± 3.05% (WM), and 78.49% ± 4.49% (combined). In contrast, the proportion of S-phase cells decreased from 42.03% ± 2.06% (control) to 25.17% ± 2.83% (γ-T3), 32.45% ± 2.68% (WM), and 20.22% ± 3.66% (combined). The increase in G0/G1 cells and decrease in S-phase cells in all treatment groups were statistically significant compared with the control group (*P* < 0.05). No significant differences were found among the γ-T3, WM, and combined groups. These results suggest that γ-T3 inhibited HeLa cell growth by arresting cells in G0/G1 phase, with WM reinforcing this effect though not significantly.

**Table 1:** Effect of WM, γ-T3, and their combination on the cell cycle distribution of HeLa cells.

Group	Cell cycle distribution (%)		
	G0/G1	S	G2/M
control	54.52 ± 1.97 c	42.03 ± 2.06 a	3.45 ± 0.10
γ-T3	72.36 ± 4.65 ab	25.17 ± 2.83 bc	2.46 ± 1.82
WM	64.21 ± 3.05 b	32.45 ± 2.68 b	3.34 ± 0.37
WM+γ-T3	75.49 ± 4.49 a	23.22 ± 3.66 c	1.28 ± 0.83

The results shown are the mean ± S.D. from three independent experiments. Lowercase letters indicate significant differences (*P* < 0.05).

### Effect of the combination of WM and γ-T3 on the apoptosis of HeLa cells

**Figure 6:**
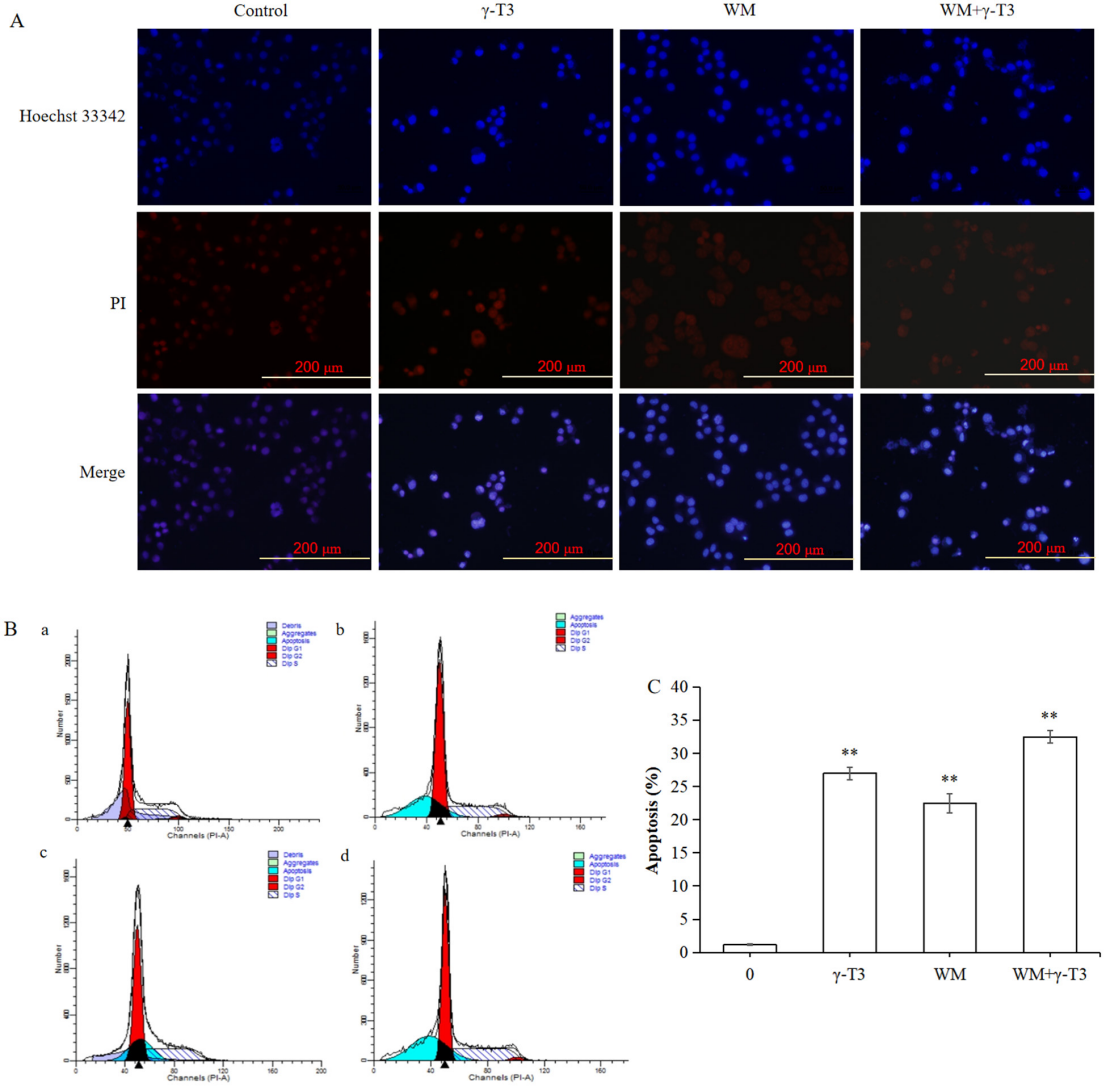
Effect of WM on γ-T3-induced apoptosis in HeLa cells. The cells were treated with WM or γ-T3 alone and in combination for 24 h. **(A)** Fluorescent microscopy images of cellular morphology observed using Hoechst 33342 and PI staining (×200). **(B)** Representative plots and **(C)** percentages measured by flow cytometry. **(a)**, blank control group; **(b)**, γ-T3 (45 μmol/L); **(c)**, WM (3 μmol/L); and **(d)**, WM (3 μmol/L) + γ-T3 (45 μmol/L). Values are presented as the mean ± SD of triplicate determinations (^**^P < 0.01; one-way ANOVA with Duncan's multiple range test).

Apoptosis of HeLa cells was assessed by Hoechst 33342/PI double staining to examine the role of the PI3K/AKT/mTOR pathway in γ-T3-induced apoptosis (Figure 6A). Cells treated with WM, γ-T3, or their combination for 24 h were also analyzed by flow cytometry (Figures 6B–C). Typical apoptotic peaks were observed in all treatment groups (Figure 6B). The percentages of apoptotic cells were 26.98% ± 0.93% (γ-T3), 22.47% ± 1.48% (WM), and 32.5% ± 0.94% (combined). All treatment groups had significantly higher apoptosis rates than the blank control group (*P* < 0.05) (Figure 6C). These findings demonstrate that γ-T3 and WM induce apoptosis in HeLa cells, with the combination showing the strongest effect.

## Discussion

It is estimated that approximately 30–40% of cancer cases may be preventable through lifestyle modifications, including dietary patterns, physical activity, and weight management (59). Phytochemicals present in fruits and vegetables have been associated with reduced cancer risk and may contribute to these protective effects (60, 61). Epidemiological studies have shown a clear association between lower cancer incidence and dietary intake of T3 (62–65). Palm oil, naturally rich in T3, has been reported to suppress chemically induced mammary tumourigenesis in female rats (66–69). While both *in vitro* and *in vivo* studies have demonstrated the strong antitumour effects of γ-T3 against various cancers, understanding its underlying mechanisms is crucial for its potential use as a functional food component and therapeutic agent.

In this study, we further examined the effects and molecular mechanisms of γ-T3 in human cervical cancer HeLa cells. Our results showed that γ-T3 at 30–60 μmol/L significantly reduced cell viability and promoted apoptosis, consistent with our previous findings (45). The IC50 value was 37.40 ± 5.70 μmol/L at 48 h, which is higher than the 18.40 ± 1.90 μmol/L previously reported at the same time point (45). In comparison, other studies reported IC50 values of ~5.0 μmol/L (5 days) (70), 7.4 μmol/L (3 days) (71), and 20 μmol/L (1 day) (72) in malignant breast epithelial cells, hepatocellular carcinoma cells, and murine melanoma cells, respectively. The relatively higher IC50 in HeLa cells may reflect differences in treatment duration, cell condition, experimental technique, seeding density, or inherent cell line characteristics.

The PI3K/AKT/mTOR signaling pathway is a key regulator of cell cycle progression, proliferation, and apoptosis, and its abnormal activation is strongly linked to tumor development (38, 73). For this reason, it has become an important target for cancer prevention and therapy (23). PI3Ks, a family of lipid kinases, are generally activated by receptor tyrosine kinases, G-protein-coupled receptors, or oncogenes such as RAS. Class I PI3Ks, which are heterodimers composed of a catalytic (110 kDa) and a regulatory (85 kDa) subunit, are most frequently implicated in cancer. PI3K activation results in AKT translocation to the cell membrane, where phosphorylation at threonine 308 and serine 473 by PDK1 and PDK2 activates AKT. Downstream of AKT, the serine/threonine kinase mTOR regulates growth, metabolism, proliferation, and migration (74). It functions as two complexes, mTORC1 and mTORC2 (75, 76). mTORC1, in particular, responds to nutrients, growth factors, and DNA damage, influencing protein synthesis and cell cycle progression (77, 78). Activated AKT stimulates mTOR directly or indirectly via phosphorylation of TSC2, which inhibits the TSC1/TSC2 complex, thereby enhancing mTOR activity (79, 80). The major downstream effectors of mTORC1 are ribosomal S6 kinase (p70S6K) and eukaryotic translation initiation factor 4E-binding protein 1 (4E-BP1) (74, 81). S6K regulates elongation factors and insulin-like growth factor 2 (IGF2), both linked to tumourigenesis (39, 82). Meanwhile, 4E-BP1 phosphorylation promotes the expression of c-Myc and cyclin D1 (83–85). c-Myc functions as a proto-oncogene regulating proliferation, growth, apoptosis, and tumor progression (86, 87), while cyclin D1 complexes with CDK4/6 to drive G1-to-S phase transition, contributing to cancer development. Cyclin D1 is frequently overexpressed in cervical, breast, bladder, and non-small cell lung cancers (88–91). Our results showed that γ-T3 treatment (30–60 μmol/L) significantly reduced both phosphorylated and non-phosphorylated mTOR, along with upstream regulators PI3K and AKT, and downstream effectors p70S6K, 4E-BP1, c-Myc, and cyclin D1. These inhibitory effects were similar to those observed with the PI3K inhibitor WM. Furthermore, γ-T3 combined with WM produced greater suppression than either agent alone, consistent with an additive or synergistic interaction. Further investigations confirmed that γ-T3 and WM inhibited proliferation, caused G0/G1 arrest, decreased the S-phase cell population, and induced apoptosis in HeLa cells, and the combination treatment enhanced these effects. Taken together, these findings suggest that γ-T3 exerts its growth-inhibitory effects primarily by downregulating the PI3K/AKT/mTOR pathway. The enhanced inhibitory effect observed following combined treatment with γ-T3 and WM does not necessarily indicate identical molecular targets but may reflect complementary modulation of signaling networks regulating PI3K/AKT/mTOR activity. Given that γ-T3 is a pleiotropic bioactive compound, it may influence upstream regulators, membrane signaling dynamics, or parallel pathways that converge on mTOR signaling. Further mechanistic studies are required to clarify whether γ-T3 directly targets PI3K or indirectly regulates pathway activity through broader cellular mechanisms.

In addition to acting as a downstream component of PI3K/AKT signaling, mTOR is increasingly recognized as a central regulator of nutrient sensing, metabolic homeostasis, and cellular stress adaptation. mTORC1 integrates growth factor and nutrient signals to regulate key anabolic processes, including protein synthesis, lipid biosynthesis, and metabolic reprogramming (92). For example, mTORC1 promotes aerobic glycolysis and lipogenesis through regulation of transcriptional factors such as HIF1α, MYC, and SREBP1 (93). In the present study, γ-T3 reduced the expression of downstream effectors p70S6K and 4E-BP1, suggesting that suppression of mTOR signaling may influence oncogenic glucose and lipid metabolism in addition to inhibiting proliferation. Previous studies have also reported that T3 may modulate cancer cell metabolism, including glycolytic pathways (94). Because mTORC1 plays a key role in ribosome biogenesis and mRNA translation via p70S6K and 4E-BP1, inhibition of these targets may contribute to reduced protein synthesis and cell-cycle progression (93, 95). Together, these findings suggest that γ-T3-mediated suppression of mTOR signaling may affect multiple aspects of tumor cell regulation, including proliferation, metabolic activity, and cellular adaptation. However, as metabolic and stress-response endpoints were not directly measured in the present study, these broader effects require further investigation.

γ-T3 exerts anticancer activity in human cervical cancer HeLa cells through suppression of the PI3K/AKT/mTOR signaling pathway. Treatment with γ-T3 (30–60 μmol/L) reduced upstream regulators PI3K and AKT, as well as mTOR, together with downstream effectors p70S6K and 4E-BP1. This inhibition further downregulated oncogenic targets c-Myc and cyclin D1, which are essential for G1–S phase transition and tumor proliferation. Functionally, γ-T3 induced G0/G1 cell-cycle arrest, reduced the S-phase population, and promoted apoptosis, thereby suppressing cell growth. The inhibitory effect of γ-T3 was comparable to WM, a PI3K-selective inhibitor. Combined treatment with γ-T3 and WM produced greater effects on pathway inhibition, cell-cycle arrest and apoptosis induction than either agent alone, suggesting an enhanced (additive) effect; formal synergy quantification would require dose–response matrix analysis. Collectively, these findings demonstrate that γ-T3 suppresses HeLa cell proliferation and survival by targeting the PI3K/AKT/mTOR cascade, highlighting its potential as a nutrition-relevant bioactive compound for further investigation.

Studies have reported that the dietary intake of T3 is relatively low (approximately 1.9–2.1 mg/person/day), which is considerably lower than the concentrations typically used in experimental studies to achieve health-promoting effects (96). In addition, a major limitation affecting the clinical application of γ-T3 is its relatively low intracellular accumulation and bioavailability, together with rapid *in vivo* metabolism, making it difficult to maintain effective circulating concentrations (97). Therefore, the concentrations used in experimental studies may reflect pharmacological rather than purely nutritional effects. Further development of more efficient and targeted delivery strategies may help improve bioavailability and tissue specificity of γ-T3.

## Conclusion

**Figure 7:**
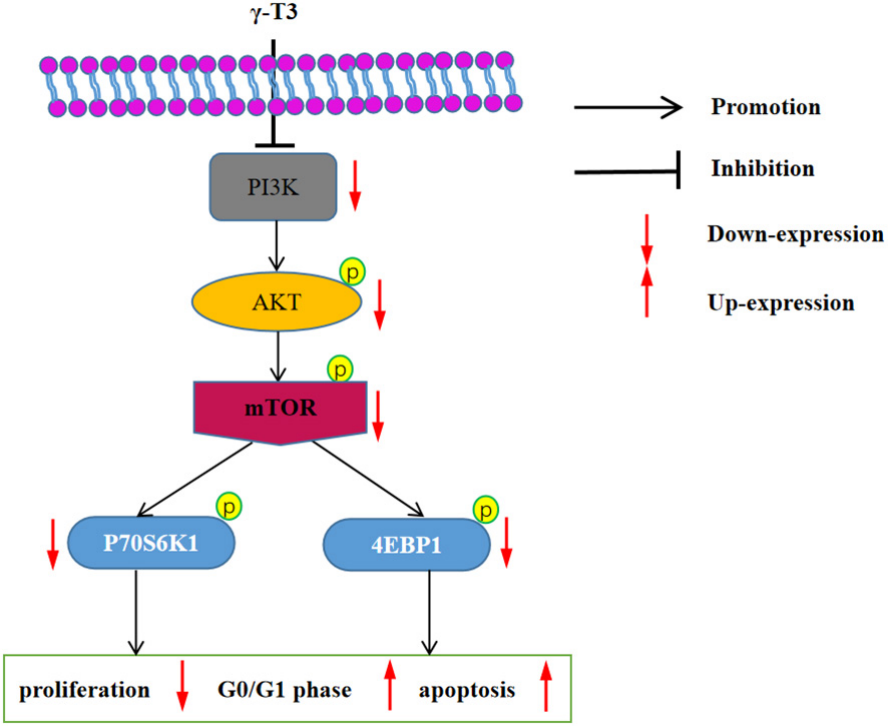
Proposed mechanism: γ-T3 inhibits HeLa cell growth by suppressing PI3K/AKT/mTOR signaling.

We demonstrate that γ-T3 (30–60 μmol/L) inhibits the PI3K/AKT/mTOR pathway in human cervical cancer HeLa cells by suppressing both upstream regulators and downstream effectors, including PI3K, AKT, mTOR, p70S6K, 4E-BP1, c-Myc, and cyclin D1. These inhibitory effects result in reduced proliferation, G0/G1 cell cycle arrest, and increased apoptosis (Figure 7). The action of γ-T3 was comparable to WM, and their combination showed enhanced efficacy, further supporting this pathway as its primary target.

Our findings indicate that γ-T3 may represent a promising therapeutic candidate for cervical cancer and could also be developed as a functional food ingredient with chemopreventive potential. Future research should validate these results in other cervical cancer cell lines and *in vivo* models, as well as assess pharmacokinetics, safety, and clinical applicability of γ-T3.

## Data Availability

The datasets presented in this study can be found in online repositories. The names of the repository/repositories and accession number(s) can be found in the article/Supplementary material.
