# Supplementary material for: γ-Tocotrienol inhibits HeLa cell proliferation likely via modulation of the PI3K/AKT/mTOR signaling pathway
Source: Front Nutr. 2026 Apr 16;13:1804277. doi: 10.3389/fnut.2026.1804277 (PMC13130507; doi:10.3389/fnut.2026.1804277)
Supplement: Supplementary file 1 [file Supplementary_file_1.doc]

**Table S1. Effect of γ-T3 on the viability of HeLa cells.**

|  | Cell viability (%) | | | | |
| --- | --- | --- | --- | --- | --- |
| group | 24 h | 36 h | 48 h | 60 h | 72 h |
| 0 μmol/L | 100.0 | 100.0 | 100.0 | 100.0 | 100.0 |
| ethanol | 104.7 | 95.4 | 92.0 | 91.0 | 89.8 |
| 15 μmol/L | 107.6 | 122.1 | 104.9 | 102.0 | 82.1 |
| 30 μmol/L | 107.6 | 99.9 | 63.8 | 51.1 | 18.0 |
| 45 μmol/L | 104.6 | 50.8 | 36.7 | 23.1 | 10.0 |
| 60 μmol/L | 90.2 | 38.6 | 26.3 | 21.5 | 9.2 |
|  | SD | | | | |
| 0 μmol/L | 0 | 0 | 0 | 0 | 0 |
| ethanol | 3.2 | 2.7 | 1.9 | 3.1 | 2.8 |
| 15 μmol/L | 4.5 | 4.7 | 6.7 | 5.6 | 6.1 |
| 30 μmol/L | 2.3 | 3.1 | 3.6 | 4.2 | 3.3 |
| 45 μmol/L | 2.7 | 3.8 | 3.7 | 2.9 | 4.2 |
| 60 μmol/L | 1.5 | 2.3 | 3.2 | 3.6 | 4.1 |

**Note:** HeLa cells were treated with the different concentrations of γ-T3 for 24, 36, 48, 60 and 72 h, and determined by the MTT method. (**Data for Figure 1B**)

**Table S2. Expression levels of PI3K, (+/-p) AKT, (+/-p) mTOR, (+/-p) p70S6K, (+/-p) 4E-BP1 c-Myc and cyclin D1 in HeLa cells**

|  | PI3K/  GAPDH | SD | AKT/  GAPDH | SD | p-Akt473/  GAPDH | SD | mTOR/  GAPDH | SD | p-mTOR/  GAPDH | SD |
| --- | --- | --- | --- | --- | --- | --- | --- | --- | --- | --- |
| 0 | 0.70 | 0.04 | 1.25 | 0.08 | 0.23 | 0.03 | 0.12 | 0.02 | 0.31 | 0.09 |
| ethanol | 0.67 | 0.04 | 1.26 | 0.08 | 0.21 | 0.04 | 0.11 | 0.01 | 0.27 | 0.08 |
| 15 μmol/L | 0.69 | 0.05 | 1.06 | 0.10 | 0.2 | 0.11 | 0.08 | 0.003 | 0.25 | 0.07 |
| 30 μmol/L | 0.59 | 0.02 | 0.95 | 0.04 | 0.18 | 0.04 | 0.06 | 0.01 | 0.22 | 0.05 |
| 45 μmol/L | 0.38 | 0.03 | 0.84 | 0.06 | 0.14 | 0.07 | 0.05 | 0.004 | 0.17 | 0.02 |
| 60 μmol/L | 0.18 | 0.04 | 0.79 | 0.03 | 0.07 | 0.02 | 0.03 | 0.01 | 0.16 | 0.02 |

|  | p70S6K/  GAPDH | SD | p-p70S6K/GAPDH | SD | 4E-BP1/  GAPDH | SD | p-4E-BP1/GAPDH | SD | c-Myc/GAPDH | SD | cyclin D1/  GAPDH | SD |
| --- | --- | --- | --- | --- | --- | --- | --- | --- | --- | --- | --- | --- |
| 0 | 1.25 | 0.13 | 0.52 | 0.04 | 0.33 | 0.04 | 0.51 | 0.05 | 0.14 | 0.02 | 0.58 | 0.03 |
| ethanol | 1.29 | 0.11 | 0.54 | 0.05 | 0.3 | 0.04 | 0.53 | 0.04 | 0.13 | 0.01 | 0.56 | 0.03 |
| 15 μmol/L | 1.24 | 0.12 | 0.49 | 0.04 | 0.31 | 0.04 | 0.53 | 0.03 | 0.12 | 0.01 | 0.61 | 0.03 |
| 30 μmol/L | 1.15 | 0.09 | 0.48 | 0.02 | 0.28 | 0.05 | 0.45 | 0.01 | 0.10 | 0.01 | 0.50 | 0.02 |
| 45 μmol/L | 1.12 | 0.1 | 0.43 | 0.03 | 0.28 | 0.04 | 0.40 | 0.03 | 0.04 | 0.01 | 0.39 | 0.02 |
| 60 μmol/L | 0.93 | 0.07 | 0.45 | 0.01 | 0.13 | 0.01 | 0.29 | 0.04 | 0.09 | 0.01 | 0.19 | 0.02 |

**Note:** HeLa cells were treated with γ-T3 at 0, 15, 30, 45 and 60 μmol/L and ethanol for 24 h. (**Data for Data for Figure 3B and D**)

**Table S3.** Effect of different concentrations of WM on cell viability.

|  | Cell viability(%) | | | | SD | | | |
| --- | --- | --- | --- | --- | --- | --- | --- | --- |
| group | 12 h | 24 h | 36 h | 48 h | 12 h | 24 h | 36 h | 48 h |
| 0 nM | 100.00 | 100.00 | 100.00 | 100.00 | 0 | 0 | 0 | 0 |
| 500 nM | 97.98 | 93.11 | 95.79 | 96.32 | 1.69 | 0.47 | 2.13 | 2.64 |
| 1000 nM | 96.52 | 90.51 | 93.11 | 93.52 | 0.55 | 0.18 | 2.34 | 2.96 |
| 2000 nM | 91.94 | 84.33 | 88.94 | 89.24 | 0.40 | 0.92 | 1.93 | 3.13 |
| 3000 nM | 89.99 | 79.32 | 84.31 | 82.84 | 0.65 | 0.49 | 2.60 | 0.36 |
| 5000 nM | 77.73 | 66.55 | 71.32 | 70.50 | 0.56 | 1.39 | 1.30 | 0.91 |
| 3‰ DMSO | 101.30 | 102.11 | 99.84 | 96.29 | 2.60 | 2.94 | 3.60 | 4.09 |

**Note:** HeLa cells were treated with 500, 1000, 2000, 3000 and 5000 nM WM for 12, 24, 36 and 48 h and examined by the MTT method. (**Data for Data for Figure 4A**)

**Table S4.** Expression levels of PI3K, (+/−p)Akt, (+/−p)mTOR, (+/−p) p70S6K, (+/−p) 4E-BP1, c-Myc, and cyclin D1 in Hela cells

| group | PI3K/  GAPDH | SD | AKT/  GAPDH | SD | p-Akt473/  GAPDH | SD | mTOR/  GAPDH | SD | p-mTOR/  GAPDH | SD |
| --- | --- | --- | --- | --- | --- | --- | --- | --- | --- | --- |
| 0 | 1.02 | 0.07 | 1.2 | 0.03 | 0.85 | 0.01 | 0.35 | 0.03 | 0.86 | 0.03 |
| γ-T3 | 0.83 | 0.06 | 0.67 | 0.06 | 0.73 | 0.01 | 0.26 | 0.02 | 0.68 | 0.02 |
| WM | 0.88 | 0.05 | 0.82 | 0.08 | 0.69 | 0.01 | 0.3 | 0.02 | 0.68 | 0.01 |
| WM+γ-T3 | 0.70 | 0.04 | 0.47 | 0.04 | 0.57 | 0.01 | 0.23 | 0.03 | 0.56 | 0.04 |

| group | p70S6K/GAPDH | SD | p-p70S6K/GAPDH | SD | 4E-BP1/GAPDH | SD | p-4E-BP1/GAPDH | SD | c-Myc/  GAPDH | SD | cyclin D1/  GAPDH | SD |
| --- | --- | --- | --- | --- | --- | --- | --- | --- | --- | --- | --- | --- |
| 0 | 1.24 | 0.07 | 1.14 | 0.03 | 1.06 | 0.07 | 2.24 | 0.04 | 0.38 | 0.01 | 0.22 | 0.01 |
| γ-T3 | 1.05 | 0.06 | 0.55 | 0.04 | 0.78 | 0.06 | 1.48 | 0.06 | 0.23 | 0.01 | 0.18 | 0.01 |
| WM | 1.04 | 0.06 | 0.57 | 0.03 | 1.00 | 0.07 | 1.79 | 0.08 | 0.28 | 0.01 | 0.12 | 0.01 |
| WM+γ-T3 | 0.78 | 0.06 | 0.47 | 0.05 | 0.59 | 0.07 | 1.05 | 0.10 | 0.18 | 0.01 | 0.08 | 0.01 |

**Note:** Cells were determined after treatment with individual or combination of γ-T3 (45 μM), WM (3 μM) for 24 h. (Data for Figure 4D and E)

**Table S5.** Effect of WM and γ-T3 on HeLa cell viability.

| group | Cell viability (%) | SD |
| --- | --- | --- |
| 0 | 100 | 1.25 |
| γ-T3 | 55.23 | 3.3 |
| WM | 78.73 | 5.8 |
| WM+γ-T3 | 40.17 | 3.6 |

**Note:** HeLa cells were treated with individual or combination of γ-T3 (45 μM), WM (3 μM) for 24 h, and examined by the MTT method. (**Data for Figure 5B**)

**Table S6.** Apoptosis percentage of HeLa cells treated with WM (3 μM) or γ-T3 (45 μM) alone and in combination for 24 h.

| group | Apoptosis(%) | SD |
| --- | --- | --- |
| 0 | 1.13 | 0.12 |
| γ-T3 | 26.98 | 0.93 |
| WM | 22.47 | 1.48 |
| WM+γ-T3 | 32.5 | 0.94 |

Note: Apoptosis was measured by flow cytometry. (**Data for Figure 6C**)

**The followings are original Image Description for Figures 3 and 4:**Figure S1 (corresponding to Figure 3A), Figure S2 (corresponding to Figure 3B), Figure S3 (corresponding to Figure 4B), and Figure S4 (corresponding to Figure 4C). The original Western blot (WB) images (Figures S1–S4) are provided in this document (from page 6) . The gels containing the protein bands were cut prior to membrane transfer to minimise the usage of membranes and antibodies. This approach has also been adopted in our previous studies, which were accepted and published in peer-reviewed journals.


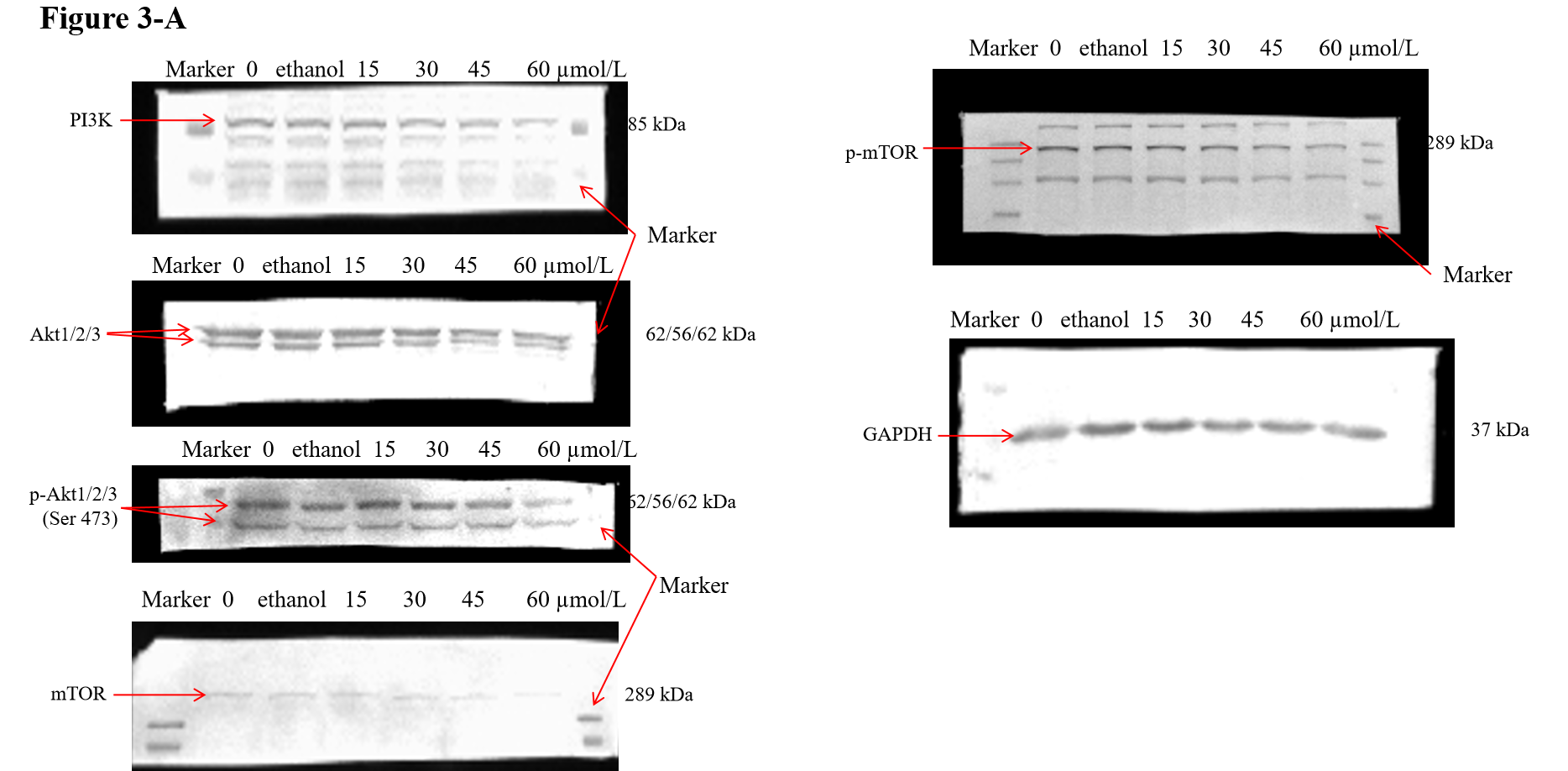


**Figure S1.** The expression of PI3K, (+/−p) AKT, and (+/−p) mTOR in HeLa cells treated with γ-T3 at 0, 15, 30, 45, and 60 μmol/L and ethanol for 24 h detected by Western blot.


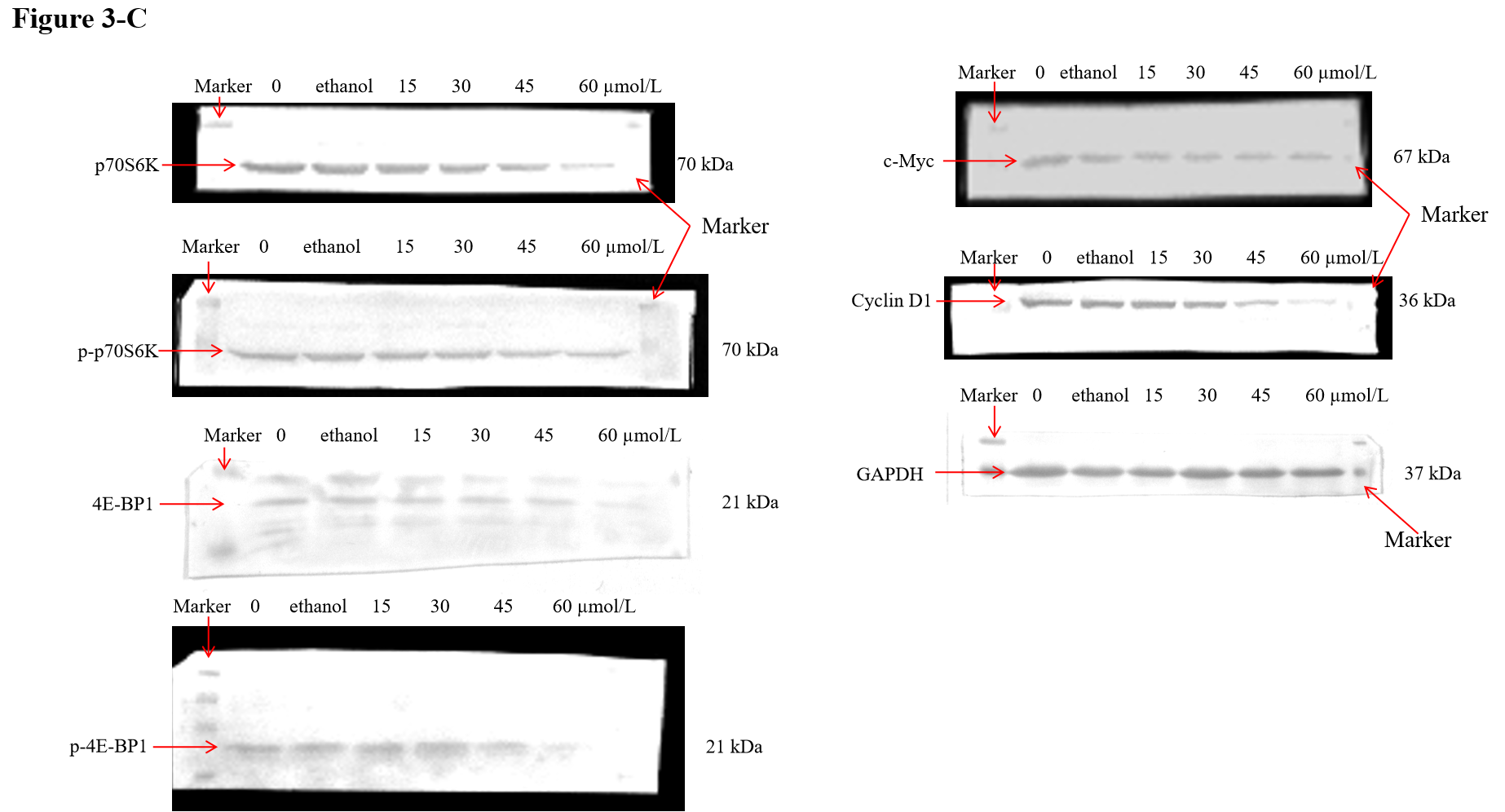


**Figure S2.** The expression of (+/−p) p70S6K, (+/−p) 4E-BP1 c-Myc, and cyclin D1 in HeLa cells treated with γ-T3 at 0, 15, 30, 45, and 60 μmol/L and ethanol for 24 h detected by Western blot.


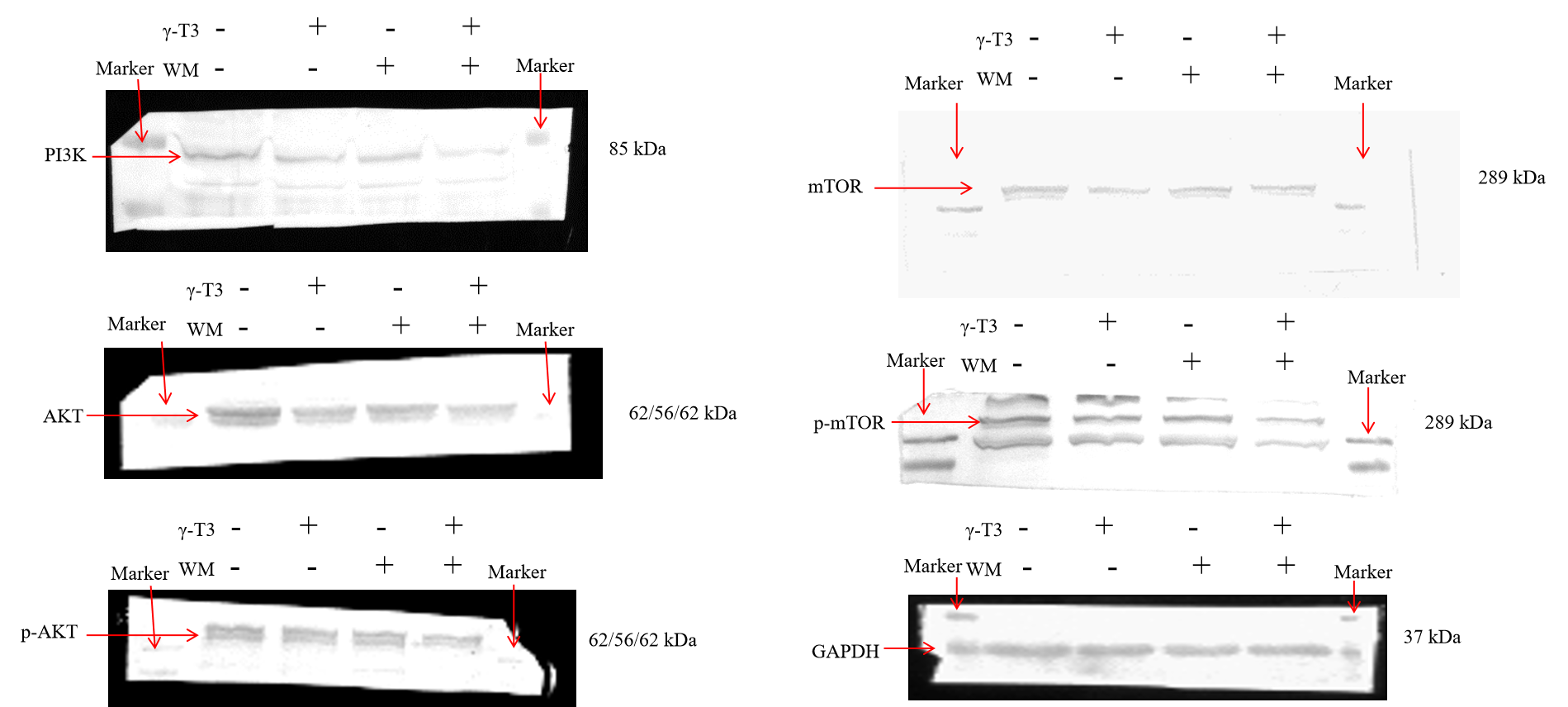


**Figure S3.** The expression of PI3K, p-Akt, Akt, p-mTOR, and mTOR in HeLa cells determined by Western blot after treatment with individual or combination of γ-T3 (45 μmol/L) and WM (3 μmol/L) for 24 h.


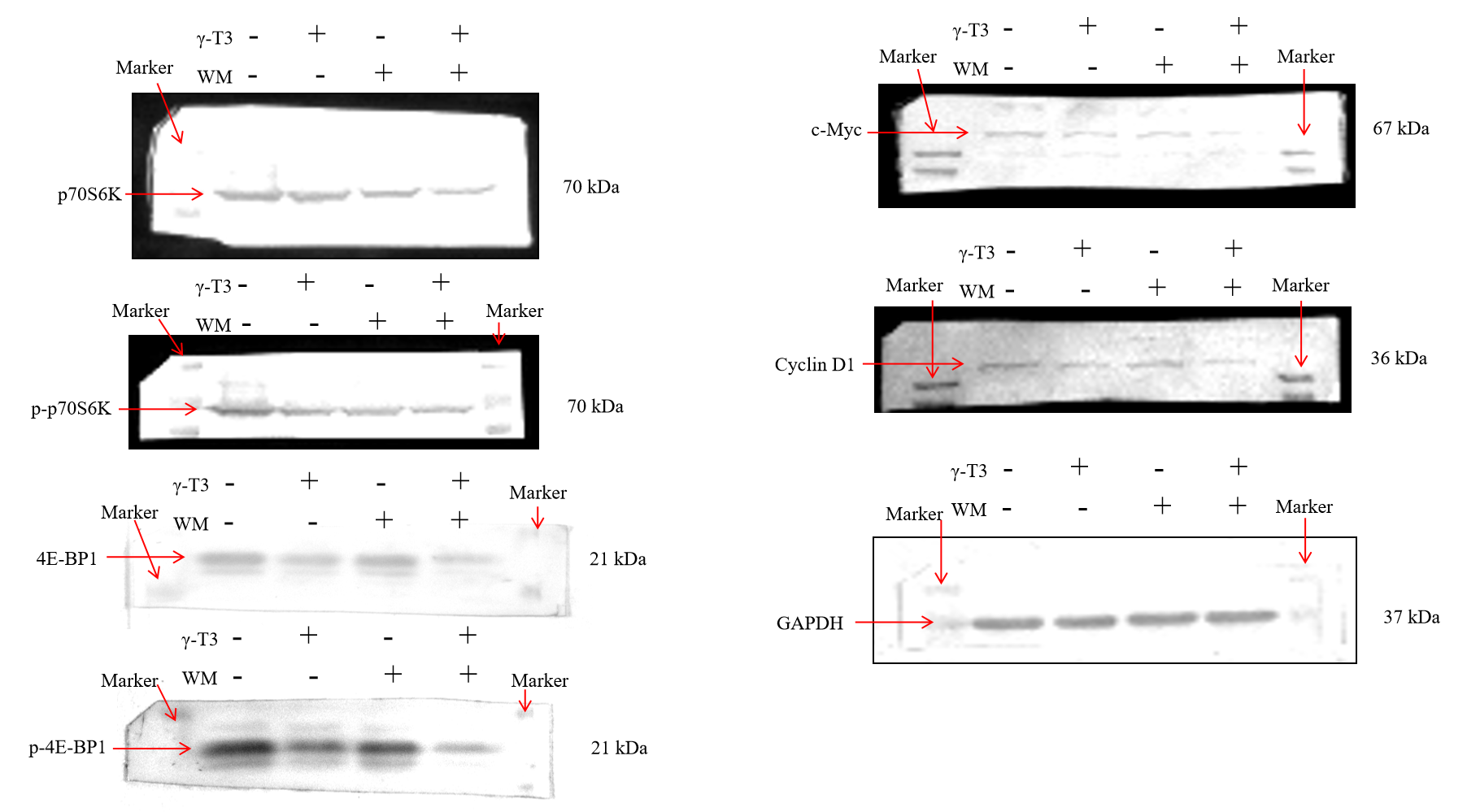


**Figure S4.** The expression of (+/−p) p70S6K, (+/−p) 4E-BP1, c-Myc, and cyclin D1 in HeLa cells determined by Western blot after treatment with individual or combination of γ-T3 (45 μmol/L) and WM (3 μmol/L) for 24 h.
